# Community-based HIV prevention research among substance-using women in survival sex work: The Maka Project Partnership

**DOI:** 10.1186/1477-7517-4-20

**Published:** 2007-12-08

**Authors:** Kate Shannon, Vicki Bright, Shari Allinott, Debbie Alexson, Kate Gibson, Mark W Tyndall, the Maka Project Partnership

**Affiliations:** 1 British Columbia Centre for Excellence in HIV/AIDS, Vancouver, Canada; 2 Faculty of Medicine, University of British Columbia, Vancouver, Canada; 3 Sex Workers United Against Violence (SWUAV), Vancouver, Canada; 4 Women's Information Safe Haven (WISH) Drop-In Centre Society, Vancouver, Canada

## Abstract

Substance-using women who exchange sex for money, drugs or shelter as a means of basic subsistence (ie. survival sex) have remained largely at the periphery of HIV and harm reduction policies and services across Canadian cities. This is notwithstanding global evidence of the multiple harms faced by this population, including high rates of violence and poverty, and enhanced vulnerabilities to HIV transmission among women who smoke or inject drugs. In response, a participatory-action research project was developed in partnership with a local sex work agency to examine the HIV-related vulnerabilities, barriers to accessing care, and impact of current prevention and harm reduction strategies among women in survival sex work. This paper provides a brief background of the health and drug-related harms among substance-using women in survival sex work, and outlines the development and methodology of a community-based HIV prevention research project partnership. In doing so, we discuss some of the strengths and challenges of community-based HIV prevention research, as well as some key ethical considerations, in the context of street-level sex work in an urban setting.

## Background

Substance-using women working in open street-level sex work markets face a myriad of health risks, including pervasive violence and assault, high rates of poverty and homelessness, drug-related harms, stigma, and social isolation [1-3]. Mortality rates among drug-using women in Vancouver, Canada, suggest a 50-fold increase as compared to the aged-matched general population, with the majority of women in street-level sex work [4]. Consistent evidence across sex work venues has documented the greatest concentration of harms among sex workers in low status, street-based, open sex work markets that frequently co-exist with open drug use markets [5,6]. Further, open sex work markets operating in criminalized prostitution environments, such as Canada, the United Kingdom and parts of Australia, are largely unregulated, and heavily policed, with high rates of violence and victimization, child exploitation, trafficking, pimping, and frequent police crackdowns [7-11].

Of particular concern, women who exchange sex for money, drugs, shelter, or other commodities as a means of basic subsistence (ie. survival sex work) have been shown to face an elevated risk of HIV transmission [12], increasingly attributed to intersections of gender-based violence, substance use, and poverty [13,14]. Evidence of the social context and gendered norms of street-entrenched sex work and drug-using populations suggest that both violence and gendered power dynamics mediate micro-risk environment and negotiation of risk reduction practices of women in both intimate and client-worker relationships [13,15,16]. Women injectors in survival sex work are also significantly more likely to required assistance to inject and to have overlapping sexual and drug use partnerships and large social networks that increase the risk of HIV infection [17,18]. Following the emergence of smokeable crack cocaine in many cities across North America, a synergistic relationship between survival sex and crack cocaine has been associated with enhanced risk for STI and HIV transmission among women, heightened violence and exploitation, and decreased control of working conditions [19-21]. In addition, women of Aboriginal ancestry continue to be highly overrepresented in new HIV infections among injection drug users [22], and constitute the majority of women working in the lowest paying tracks across Canadian cities [23].

In Vancouver, Canada, a city drug policy response, known as the Four Pillars strategy, and several innovative harm reduction efforts have been shown to be highly successful in reducing the harms of drug users, including primary and secondary prevention, extensive fixed and mobile syringe exchange programs, a heroin maintenance trial and two supervised injection facilities [24,25]. Yet there continues to lack a systematic response to the alarming rates of violence and health-related harms faced by substance-using women in street-level sex work in this setting [26]. The absence of a gender-specific harm reduction and prevention efforts is particularly noteworthy given the ongoing violence and victimization of sex workers in this setting [27,28], including a highly profiled case of murdered and missing women. Furthermore, evidence both locally and internationally suggests that women in sex work are consistently less likely to utilize conventional health and HIV service models due to lack of access, restrictive hours, absence of women specific services, high levels of stigma and concerns of privacy and disclosure [29-31].

In an effort to respond to existing gaps in prevention and policy, a community-based HIV prevention research project was developed to investigate the health-related harms, service barriers, and impact of current harm reduction and prevention strategies among women working in survival sex work in Vancouver, Canada. This paper outlines the development and methodology of the Maka Project Partnership, and discusses some of the strengths and challenges of community-based HIV prevention research, as well as ethical considerations, in the context of survival sex work in an urban setting.

## Methods

### Development of CBR Partnership

An initial gap in service access, HIV prevention and harm reduction for survival sex workers was identified as a key issue through informal conversations between health providers, staff, and sex workers at an inner city drop-in centre. In operation since 1987, Women's Information Safe Haven (WISH) Drop-In Centre Society connects with an estimated 200 women engaged in survival sex work per night. While the mandate is not exclusive to Aboriginal women, over half of the women that come through its doors are of First Nations, Metis and Inuit ancestry. The project works closely with WISH's well-established Aboriginal Health and Safety Project for Women in the Sex Trade (AHIP), as well as other key Aboriginal and sex work collaborators.

In 2004, researchers were approached to collaborate on an initial needs assessment of women attending the drop-in. The results led to the conception and design of both a research and a service arm [29]. The service arm focuses on peer outreach, resource development, and ongoing wellness nights at the drop-in that help to support the knowledge translation activities of the research arm. The CBR project partnership, initiated in late 2005, was developed and continues to be supported through active consultation and exchange of information between researchers and community. The community co-investigators represent sex work, Aboriginal, and youth service organizations. The research is guided by a sex-for-work perspective and adheres to participatory-action research methodologies [32,33]. In particular the project is guided by the OCAP principles of ownership, control, access and possession initially developed by the First Nations' Governance Committee and subsequently adopted by the Canadian Aboriginal AIDS Network (CAAN). Providence Health/UBC Ethics Review Board provided ethical approval for this study. In addition, PACE (Prostitution Alternatives, Counselling and Education (PACE) Society) Policy Group provided community ethics review from a sex work research and policy perspective and the project adheres to these best practices [34].

### Peer Involvement

A key component of the project is capacity-building among a team of women in survival sex work, supported by an open Community Advisory Board (CAB), that inform all stages of the project. Initial CAB tasks were to identify the working role of the CAB and develop a hiring process for peer team of women. The hiring process aimed to ensure a transparent process, including extensive and flexible options for informing and inviting women to contact the project (outreach, community agency visits, open house for women) and the creation of a community-peer hiring panel (CAB members). Through this process, a team of women with a lived experience of survival sex work were hired, trained and support to play an active role in guiding, developing and conducting the research. These positions are low-threshold employment positions that work from a harm reduction perspective, similar to models of other sex work and drug-user groups. There is considerable focus on capacity building and training, as well as ongoing support and referral for addictions counselling, drug treatment, supportive housing, and child care. Extensive training modules were conducted in collaboration with local community agencies and sex work groups, including sex work specific training created and conducted by PACE Society on lateral oppression, vicarious trauma, debriefing and conflict resolution; participatory-action research principles, methodologies, ethics, and informed consent; and health, HIV/HCV prevention, and harm reduction conducted by community health providers, sex work groups, and Aboriginal agencies.

### Multi-Research Methodologies

In keeping with community-based research principles, this project adopts multiple research methodologies, including ongoing qualitative focus group discussions, social mapping, and a prospective cohort (6 monthly interview questionnaires and HIV screening) over a three-year period. All focus group discussions are facilitated or co-facilitated by a member of the peer team and inform the 'lived experiences' of survival sex work and barriers to and facilitators for prevention and harm reduction efforts. Individual informed consent is obtained prior to the discussion group and verbal rather than written consent is provided at the time of the interview, due to participant concerns of confidentiality. Discussion groups last approximately two hours and all participants receive Can$25 compensation for their expertise and time.

### Mapping

A particularly novel component of the project is the social mapping, a participatory-action research tool that facilitates community access to hidden populations and highlights local expertise. Initial piloting of the maps with over 60 women facilitated by the Maka peer team has informed subsequent recruitment and outreach efforts for both research and service arms of this project. In particular, women are provided with a map of Vancouver's Downtown Eastside and surrounding communities and asked to mark 1) strolls where they work and live; 2) current working conditions (lighting, phones); 3) high and low risk areas for violence and bad dates; 4) working areas impacted by police presence and harassment; 5) areas of health and syringe availability and disposal.

### Time-Space Sampling

Given the difficulty in accessing a representative sample of sex workers due to the illegal and clandestine nature of sex work and unknown boundaries of this population [35], mapping and time-space sampling strategies were used to enhance attempts to obtain a representative sample of survival sex workers, supported by standard outreach recruitment strategies used among street-involved populations. Time-space sampling strategy is a probability-based method used to enrol members of a hidden population at times and places where they congregate rather than live [36,37], with physical spaces (such as bars, parks, or sex work strolls) rather than persons as the primary sampling unit. Although time-space sampling has been primarily used with MSM populations in gay venues [36,37], the adaptation of this strategy to sex work research is promising. Based on sex work strolls identified through the ongoing mapping in 2006, the peer outreach team conducted systematic outreach during staggered working hours (late night, early morning and daytime) and locations (sex work strolls) to invite women to participate. A researcher or nurse accompanied the outreach team during the late night outreach hours to ensure safety through use of a vehicle and facilitate mobile outreach to more dispersed areas. Staggered days of the week as well as times of the month were used to ensure as representative a sample as possible. Following initial recruitment, the majority of women were invited to participate in the interviews the following day at the project office, an area close to several of the strolls. Based on youth consultations and identified barriers among younger women, specific community drop-in and commercial spaces (such as corner stores, coffee shops) were identified close to the strolls to conduct the interview questionnaires with youth. As well, if business was slow, women conducted the interviews during these late night hours and the worker, along with the Maka outreach team and nurse, would chose a safe and private location.

### Prospective Cohort

Through time-space sampling, a total of 205 women were initially invited and agreed to participate in a baseline visit over a six month period in 2006 (response rate of 93%), with ongoing 6 monthly follow-up visits scheduled to continue through 2008. Baseline and follow-up include detailed interview questionnaires administered by the peer interviewers and HIV screening by the project nurse, supported by extensive pre and post-test counselling. Women 14 years of age and older who have used illicit substances (not including marijuana) within the last month and are actively engaged in street-level sex work are eligible to participate. Semi-structured interview questionnaires elicit responses related to current and past experiences of sex work, violence and trauma, health and addition service access, working conditions, and sexual and drug-related harms. HIV screening is completed by the project nurse using the INSTI rapid HIV test (Biolytical, Vancouver), and new reactive tests are confirmed by western blot. A pre-test counseling questionnaire is completed by the project nurse on detailed questions relating to overall health, experiences with HIV and Hepatitic C testing, and current and past abuse experiences in order to facilitate counselling and referral to support services. Participants receive Can$25 compensation at baseline and each follow-up visit.

## Results & Discussion

Sex work researchers have described some of the key ethical issues and challenges in conducting non-exploitative research in this population both in Canada and internationally [34,35,38-40]. Of particular concern has been the lack of sampling frame due to the unknown size and boundaries of sex work populations, concerns of privacy and confidentiality due to stigma and illegality of sex work and drug use, and finally, dichotomies of sex work and victimization that precipitate misconceptions of sex work industry as homogenous [35]. Through the development of this CBR partnership and methodology, several additional strengths and challenges emerged, as well as ethical issues, related to HIV prevention research with survival sex workers in this setting that are of important consideration.

### Privacy and Confidentiality

The concern of ensuring privacy and confidentiality of participants requires particular consideration in the sex work context, in addition to raising some key ethical issues related to HIV prevention research. Given the historically oppressive nature of research of sex work, significant concern from the onset by both the community partner and sex work activists was that the research did not further stigmatize a highly marginalized population of women, or falsely precipitate sex workers as "vectors of disease". A concern similarly voiced in other settings across the globe [39,41]. As such, the involvement of women in sex work and the community partner organization in all aspects of the research, from conception and design, to interpretation and dissemination of results, is a core aspect of ensuring accountability of the research.

### Ethical Issues

Two key ethical issues emerged of important relevance in HIV related research with sex work populations. First, while HIV is a reportable illness using the standard ELISA test, the new rapid point of care test provides women with the opportunity to receive anonymous HIV screening. Women are advised during extensive pre-test counselling that the INSTI test is not a diagnostic test, and a reactive test needs to be confirmed by a western blot, as per standard of care. Women have the choice of being referred to a physician for follow-up testing, or completing the follow-up testing at the Maka Project office. Early findings suggest increased acceptability and utilization of point of care testing among a highly marginalised population of women, with 96% of women agreeing to HIV screening at first visit. Within this context, the role of HIV reportability and disclosure in survival sex work needs to be explored [34], particularly given growing reports of the criminalization of non-disclosure in the Canadian setting[42].

Secondly, reporting of violence experienced by youth less than 18 years of age is a major limitation to research with legal minors [43], and has been previously described in this setting among at-risk, street youth [44]. A large number of youth (under 18 years of age) are involved in survival sex work, with early research from this project identifying a median age of sex initiation of less than 18 years. Of particular concern, initiation of sex work during adolescents (less than 18 years) was independently associated with a two-fold increased odds of baseline HIV infection [45]. It is therefore crucial that sex work research engages adolescent and young women in the research during these critical time periods and identifies HIV prevention strategies tailored to this population. However a requirement by law is that any disclosure of violence among those less than 18 years of age must be reported to health authorities, including exploitation of a minor, thus placing the duty to report on researchers. Given the difficulty in engaging this population and the high rates of violence known among women and youth in survival sex work, this represents a substantial limitation. Further exploration of youth reporting requirements from a policy perspective is warranted, particularly as it relates to sex work and exploitation of minors.

### Participatory-Action Research Process

Consistent with recent literature on public health partnerships [46], the CBR research project was developed through a process of co-construction of knowledge in the negotiated space of sex workers, community and the academic partners. This negotiated space is a process inherent in participatory-action research and public health partnerships with marginalized populations as it seeks to confront and reduce power imbalances. Evidence suggests that this transdisciplinary dialogue can propose new ends to public health, rather than applying standardized solutions to health disparities by outside experts [46].

However, a particular challenge to the CBR process in this community has been the balancing of interests of academic and community partners, as well as survival sex workers themselves. Three sets of partners are represented in the research: a service agency for sex workers, survival sex workers, and the researchers. Emerging discussions in community-based HIV prevention research have focused on the challenges of defining a "community" in the CBR process [47]. In health, community often refers to individuals who share cultural, social or economic ties and a physical space [48], while others suggest a broader definition of common culture, social structure and awareness of an identity as a group. A particular concern from the onset of this project was that those working in service organizations do not necessarily reflect the voice of women on the street. Sex workers working in service organizations may have different access to resources than those who struggle daily with poverty and addictions, and as such, may have different perspectives on prevention needs. In order to ensure equal representation, our CAB includes at least equal number of survival sex workers. In addition, similar to other peer-based organizations in this community, women were hired into low threshold employment allowing those with survival sex work experience, a voice seldom heard in sex work activism, to play an active role in guiding the research process.

In addition to the many strengths of ensuring active involvement of those currently involved in survival sex work for both sex workers and researchers [38,39,49], there were several challenges that emerged in terms of sustainability of low threshold employment positions for women, and time-investment in the process. The inherent need for significant flexibility in structure of peer positions that support women living with addictions and poverty presented challenges in sustainability and resources focused on training and support. Ongoing challenges with women in and out of corrections, detoxification and drug treatment, as well as issues of housing, childcare and addictions play a constant role in carrying out the project. For many women, their dual roles as researchers and participants constitutes both a positive experience, as well as a challenge. In particular, the shift in their positions within the street-level sex work community presented unique considerations in terms of protection of confidentiality and privacy as professionals. As well, the potential for issues, such as violence, to trigger women during the research process was of ongoing consideration. Extensive training carried out by sex work agencies and health providers, weekly project staff meetings, including a check-in and out, as well as ongoing debriefing sessions help facilitate and ensure a positive experience for both sex workers and researchers. Further, consideration of increased allocation of research resources for capacity-building and peer support will help to sustain the effectiveness of CBR partnerships.

## Conclusion

High rates of health and drug-related harms, including violence and victimization, persist among women in open street-level sex worker markets in cities across Canada, and globally [27,28,50,51]. Yet despite a highly publicized HIV epidemic and ongoing prevention and harm reduction interventions targeting injection drug users in this setting, there remains a clear lack of policy and interventions tailored to promoting the health and safety of substance-using women working in survival sex work. As such, this community-based HIV prevention research partnership is well situated to inform evidence-based prevention and policy reforms that are responsive to the needs of this population. Furthermore, the development and methodology of this CBR partnership offers important insight into the strengths and challenges, as well as ethical considerations, of community-based HIV prevention research among substance-using women who engage in survival sex work in an urban setting.

## Authors' contributions

KS conceptualized the manuscript, wrote the original draft, and incorporated suggestions from authors into the final version of the manuscript. VB, SA, DA, KG and MWT were involved in conception of the methodology and provided critical feedback on content and revisions to the original draft. All authors read and approved the final version of the manuscript.
